# Iodine Intakes of Victorian Schoolchildren Measured Using 24-h Urinary Iodine Excretion

**DOI:** 10.3390/nu9090961

**Published:** 2017-08-30

**Authors:** Kelsey Beckford, Carley A. Grimes, Claire Margerison, Lynn J. Riddell, Sheila A. Skeaff, Caryl A. Nowson

**Affiliations:** 1 Institute for Physical Activity and Nutrition, Deakin University, Locked Bag 20000, Waurn Ponds, Geelong, VIC 3220, Australia; kbec@deakin.edu.au (K.B.); carley.grimes@deakin.edu.au (C.A.G.); claire.margerison@deakin.edu.au (C.M.); lynn.riddell@deakin.edu.au (L.J.R.); 2 Department of Human Nutrition, University of Otago, 362 Leith St, North Dunedin, Dunedin 9016, New Zealand; sheila.skeaff@otago.ac.nz

**Keywords:** iodine, urine, fortification, schoolchildren, Australia

## Abstract

Mandatory fortification of bread with iodized salt was introduced in Australia in 2009, and studies using spot urine collections conducted post fortification indicate that Australian schoolchildren are now replete. However an accurate estimate of daily iodine intake utilizing 24-h urinary iodine excretion (UIE μg/day) has not been reported and compared to the estimated average requirement (EAR). This study aimed to assess daily total iodine intake and status of a sample of primary schoolchildren using 24-h urine samples. Victorian primary school children provided 24-h urine samples between 2011 and 2013, from which urinary iodine concentration (UIC, μg/L) and total iodine excretion (UIE, μg/day) as an estimate of intake was determined. Valid 24-h urine samples were provided by 650 children, mean (SD) age 9.3 (1.8) years (*n* = 359 boys). The mean UIE of 4–8 and 9–13 year olds was 94 (48) and 111 (57) μg/24-h, respectively, with 29% and 26% having a UIE below the age-specific EAR. The median (IQR) UIC was 124 (83,172) μg/L, with 36% of participants having a UIC < 100 μg/L. This convenience sample of Victorian schoolchildren were found to be iodine replete, based on UIC and estimated iodine intakes derived from 24-h urine collections, confirming the findings of the Australian Health Survey.

## 1. Introduction

Mild iodine deficiency in children has been associated with impaired growth [1] and reduced intellectual capacity [2,3]. Monitoring of iodine nutrition in Australia prior to the 21st century was scarce, and was limited to very small sample sizes [4,5,6,7,8]. Prior to the 1990s Australia was believed to be iodine-sufficient, with the exception of the island of Tasmania where endemic goitre was prevalent until the introduction of an iodine supplementation program in 1950 [4,9]. This, in conjunction with the serendipitous contamination of milk by newly-introduced iodine containing sanitisers in the dairy industry, saw a dramatic increase in iodine intakes in the early 1960s [4,6,7,9]. Both Tasmania and mainland Australia were subsequently considered iodine replete using spot urine samples, however, a study conducted in Sydney in the late 1990s reported iodine deficiency in a small sample of pregnant women [10] and studies reported iodine deficiency in Tasmanian school children in 1998 and 2000 [8]. Following this, a number of studies documented iodine deficiency in Australian children [11,12,13,14,15] and adults [16,17,18,19,20]. In order to combat the re-emergence of deficiency, mandatory fortification of all commercially-produced yeast-leavened bread, excluding organic bread, with iodized salt was implemented in 2009 [21]. Studies conducted post-fortification utilizing spot urine samples, including the 2011–2013 Australian Health Survey (AHS), have reported an improvement in iodine status among children and adults, when compared to pre-fortification [22,23,24,25,26].

The most common method for monitoring the iodine status of a population is urine samples, as approximately 90% of ingested iodine is excreted in the urine within 24 h [27,28,29], with a median urinary iodine concentration (UIC) ≥100 μg/L measured in spot urine samples indicative of sufficiency [30]. Due to the considerable variation in iodine excretion over the course of the day [31,32,33,34], spot samples are not able to provide an estimate of the actual iodine intake of a population [35,36], however the use of a 24-h urine sample to determine urinary iodine excretion (UIE) in μg/day allows for a good estimate of recent iodine intake [37,38,39,40,41]. Whilst the AHS was able to estimate iodine intakes using 24-h food recalls [42], dietary assessment methodologies are not considered as accurate as urine samples for estimating iodine intakes [43]. Therefore, the use of a single 24-h urine sample is able to provide a more objective measure of actual iodine intake.

Therefore the aim of the following study was to determine daily total iodine intake of a sample of Victorian schoolchildren on one day utilizing 24-h urinary excretion of iodine and to assess differences across age, gender, and socioeconomic status.

## 2. Materials and Methods

### 2.1. Study Design

The data for this study was taken from the Salt and Other Nutrient Intake in Children (SONIC) study, a cross-sectional study conducted in a convenience sample of Victorian primary schools from June 2010 to May 2013. The detailed protocol for the SONIC study has been previously published [44]. Ethics approval was obtained from the Deakin University Human Research Ethics Committee (project no. EC 62-2009). Informed consent was obtained from both the child and their primary caregiver.

### 2.2. Measures

Fifty-six schools participated in SONIC, and consent was obtained from 852 children; 41 children withdrew from the study, 25 children did not agree to attend an off-school campus data collection day, six children were aged >13 years, resulting in a final sample size of 780 participants. A demographic questionnaire was completed by the child’s primary caregiver, including questions about the child’s date of birth, sex, birth weight and other health information. The questionnaire also collected information on the highest level of education attained by the child’s primary caregiver, as a measure of socio-economic status (SES). The use of parental education as a marker of SES has consistently been used in dietary studies in Australian children and adolescents [45,46,47]. SES was defined as: (i) high: includes those with a university/tertiary qualification; (ii) mid: includes those with an advanced diploma, diploma, or certificate III/IV or trade certificate; and (iii) low: includes those with some or no level of high school education. As this data was not available for all participants (*n* = 554, 85%), an alternative measure of SES was also used. Socio-Economic Indexes for Areas, Index of Relative Socio-Economic Disadvantage [48], was used to group participating schools, based on school postcode, into tertiles of socio-economic disadvantage. This marker was used to define socio-economic status, whereby the participant was grouped as either low, mid or high SES depending on the tertile of the school they attended.

### 2.3. Anthropometric Measurements

Height and weight were measured by trained research staff following standard protocols [44]. BMI values were converted to age- and sex-adjusted BMI z-scores using the 2000 US Centers for Disease Control and Prevention growth charts [49,50]. Participants were grouped into weight categories (underweight, healthy weight, overweight, obese) using the International Obesity Taskforce BMI reference cut-offs for children [51,52].

### 2.4. 24-h Urine Collection

Children could elect to complete the urine collection on either a school day (commencing at approximately 09:00 Monday to Friday) or a non-school day (i.e., weekends, public holidays, or school holidays).

Both the parent and child were carefully instructed on the correct collection protocol, and were provided with written instructions. At the commencement of the 24-h urine collection, children were instructed to empty their bladder, discard this urine and note this as the start time. Following this, all urine voided was collected up until the corresponding 24-h finish time. The start and finish time, along with any missed collections and/or spillages, were recorded on a urine collection slip by the child’s primary caregiver, which was returned with the completed urine sample. Following measurement of urine volume and analysis for urinary sodium and creatinine by a commercial pathology laboratory (Dorevitch Pathology), 2 × 10 mL aliquots were taken per participant for storage and stored at −80 °C. 

### 2.5. Urinalysis

Urinary iodine concentration (UIC, μg/L) was determined using a modification of the method of Pino et al. [53] at the Department of Human Nutrition, University of Otago, Dunedin. The internal standard used was a pooled urine sample (mean (SD) iodine concentration 84(4) μg/L) which gave a CV of 4.5% (*n* = 54). Seronorm (Seronorm Trace Elements Urine, Sero As, Stasjonsveien, Billingstad, Norway) was used as an external standard, giving a mean (SD) iodine concentration of 131 (1) μg/L (expected range 131–150 μg/L) and a CV of 1.01% (*n* = 54). urinary iodine excretion (UIE, μg/24-h) was calculated using the following equation [28,30]:

(1) $$\mathrm{UIE}\;\left(\mu g/24\;h\right)=\frac{\mathrm{UIC}\;\left(\mu g/L\right)\times 24\;h\;\mathrm{urine}\;\mathrm{volume}\;(L/24\;h)}{0.92}$$

Urinary creatinine concentration was assessed using the Jaffe reaction [54] on the Siemens Advia 2400 analyzer (Siemens Healthcare, Bayswater, Victoria, Australia), with a CV of 3–25%.

### 2.6. Validity of Urine Samples

Urine samples were considered incomplete if collection time was <20 h or >28 h (*n* = 5), total volume was <300 mL (*n* = 37), the participant reported missing >1 collection (*n* = 14) or urinary creatinine excretion was less than 0.1 mmol/kg body weight/day (*n* = 69), leaving 667 participants with a valid urine sample [55]. If the duration of the collection was not exactly 24 h but within 20–28 h, urinary electrolytes, creatinine and total volume were standardized to a 24-h period (i.e., (24 h/urine duration (h)) × urinary measure). Due to lab processing errors in the original SONIC study, 17 participants did not have aliquots of urine stored for analysis. Therefore, 650 participants (86%) had a stored aliquot available for iodine analyses. Data for these 650 participants has been reported in the present analysis.

### 2.7. Statistical Analysis

All analyses were completed with STATA/SE 14.0 software (StataCorp LP, College Station, TX, USA), and a *p* value < 0.05 was considered statistically significant. For analysis children were grouped into age groups (4–8 years and 9–12 years) that are consistent with the National Health and Medical Research Council (NHMRC) Nutrient Reference Values for Australia and New Zealand reference age groups [56]. In order to make comparisons with the Australian Health Survey (AHS) results [26], we have also presented results for the subgroup of participants within the age range of 5–11 years.

Descriptive statistics (mean values and standard deviations or proportions and numbers) were used to describe participant characteristics, stratified by gender. Normality of continuous variables was assessed using box plots, histograms and the Shapiro-Wilk test. UIE (μg/24-h), UIC (ug/L), creatinine (mmol/24-h), and volume (L/24-h) were expressed as mean (SD) as they were determined to be sufficiently normally distributed. As UIC (μg/L) is commonly expressed as median (IQR), we also report this to enable comparisons to the literature. Differences in the proportion of participants by gender and across sociodemographic characteristics was determined using *χ^2^* tests.

Multiple linear regression, with adjustment for school cluster was used to assess differences in urinary parameters between age groups and socioeconomic groups. Linear regression, adjusted for school clustering, was used to assess differences across socioeconomic groups (adjusted for age and gender). The unstandardized beta co-efficient (β) has been presented for both the adjusted and unadjusted models. The underlying assumptions of linear regression (i.e., homoscedasticity, normality) were confirmed using regression plots of residuals.

To assess the adequacy of UIE compared to dietary recommendations, the proportion of the population with a UIE below the age and gender specific estimated average requirement (i.e., 65 μg/day for 4–8 year olds and 75 μg/day for 9–12 year olds) [56] was determined using the EAR cut-point method [57] and expressed as *n* (%), and Chi-square (χ^2^) test used to assess the difference in proportions between age and gender groups exceeding the EAR. The proportion of the population falling below the recommended UIC of 100 μg/L and 50 μg/L was expressed as *n* (%), and *χ^2^* test used to assess the differences in proportions between age and gender groups. 

## 3. Results

### 3.1. Demographic Characteristics

Overall 55% of children were male, the average age was 9.3 years and 62% and 61% were of high socio-economic background based on school postcode SEIFA and primary caregiver education index, respectively (Table 1). In total, 17% of children were either overweight or obese and more than half completed the 24-h urine collection on a non-school day.

### 3.2. Urinary Parameters, by Gender

The mean (SD) UIE and median (IQR) UIC for all participants were 104 (54) μg/24-h and 124 (83, 172) μg/L, respectively (Table 1). Seventy-three percent of participants had a UIE greater than the recommended EAR for their age and gender group [56]. The proportion of participants falling below the World Health Organization (WHO) recommendation of 100 μg/L based on UIC [30] was less than 50%, with only 8% of the population below 50 μg/L.

Overall, males had higher UIE and UIC, compared to females (*p* < 0.01), and more females had a UIE below the recommended age-specific EAR [56]. In addition, a higher proportion of females fell below the WHO recommended UIC cut-offs of 50 μg/L and 100 μg/L [30] (Table 1).The mean UIE of participants classified as having a UIC below 50 μg/L (*n* = 49) was 42 (26) μg/day and 73 (38) μg/day for those with a UIC below 100 μg/L (*n* = 233).

### 3.3. Differences in Urinary Parameters, by Age

A 1 year increase in age was associated with a 6 μg/24-h increase in iodine excretion and accounted for 4% of the variance in UIE (*R*^2^ = 0.04, β(SE) = 5.9 (1.4), *p* < 0.001). This association remained significant after adjustment for gender (*R*^2^ = 0.07, β = 5.7 (1.4), *p* < 0.001).

Children in the 9–12 year age group had a 15% higher mean UIE compared to the 4–8 year old participants (Table 2, *p* < 0.001). There was no significant difference in UIC or the proportion of participants falling below 100 and 50 μg/L between the two age groups, nor was there a difference in the proportion of participants with a UIE meeting the age-specific EAR.

When broken down into the AHS age group of 5–11 year olds (*n* = 617), the median (IQR) UIC was 124 (83, 172) μg/L and mean (SD) UIC and UIE were 133 (67) μg/L and 102 (52) μg/24-h, respectively. The proportion of 5–11 year old participants with median UIC below 50 and 100 μg/L were 36% and 7%, respectively.

### 3.4. Urinary Parameters, by SES

There was no significant effect of SES based on school postcode on UIC, UIE, or the proportion meeting the recommendations for EAR or UIC cut-offs (Table 3). When SES was based on primary caregiver education there was no change in these findings (data not shown).

## 4. Discussion

The present study is the first to examine the iodine intakes of Victorian schoolchildren, using 24-h urine samples. The mean UIE of both age groups exceeded the age-specific estimated average requirement (EAR) indicating that, overall, the iodine intakes of this group of schoolchildren are sufficient. These data are supported by the observation that the median UIC of 124 μg/L exceed the recommended 100 μg/L set by the World Health Organization (WHO) indicative of adequate iodine status. We found that age and male gender were significant predictors of UIE, albeit minimally, and this is most likely due to increased overall food intake. There was, however, no difference in UIE between the different socioeconomic groups. These findings, along with those of other studies conducted post-fortification of bread with iodized salt [25,58], provide evidence that compared to pre-fortification data Australian schoolchildren are now iodine replete. Therefore, it appears that the iodine intakes of Australian schoolchildren have increased to sufficiency since the introduction of mandatory fortification of bread with iodized salt.

The mean UIC of 5–11 year olds in our study, as determined using 24-h urine samples, was 124 μg/L compared to 178 μg/L in the same age group assessed by spot urine samples in the Australian Health Survey (AHS), with a higher proportion of participants falling below the recommended cut-off of 100 μg/L when compared to the AHS (36% vs. 20%) [59]. It is not unusual to observe lower iodine excretion rates in Victoria, when compared to the rest of Australia, with studies conducted both pre- and post-fortification observing significantly lower iodine excretion amongst Victorians [4,14,15,26,60]. In the AHS in particular, Victorians aged 18 years and over had a 10% lower median UIC compared to the Australian average (113 vs. 124 μg/L) as well as 11% more participants with a UIC below 100 μg/L (42% vs. 37%) [26]. Reasons for the regional variation in iodine intakes within Australia are not well established, but could be related to possible regional variations in the iodine concentration of soil, milk, and differences in water iodine levels [61].

Another reason for the differences between the results in our study and those of the AHS may be due to the differences in the urinary methodologies. Whilst the AHS utilized spot urine samples, we measured UIC in 24-h urine samples [31,33]. Previous studies comparing 24-h UIC and spot UIC in schoolchildren [35,62] have found that spot UIC tends to be lower than 24-h UIC, by approximately 10% [35,62]. Conversely, the 24-h UIC observed in the present study was 30% lower than the spot UIC observed in the AHS. This could be due to the timing of the spot samples in the AHS compared to previous studies, as it has been demonstrated that spot UIC can vary significantly depending on the timing of the sample collection [33,34,63,64]. One study in 60 Brazilian adults observed a 30% lower UIC in morning spot samples, compared to overnight samples (183 vs. 253 μg/day, *p* < 0.001) [34]. It is important to note that the studies comparing spot and 24-h UIC in schoolchildren utilized first morning spot samples [35,62] whereas the AHS did not specify a time for the spot urine collection [42]. Therefore the urine samples in the AHS could have been collected overnight when iodine excretion is highest, and this might explain the higher UIC observed in the AHS, when compared to the 24-h UIC observed in the present study.

A recent systematic review of studies comparing spot and 24-h urines for estimating the iodine intake of a population concluded that there is currently not enough evidence to determine whether UIC estimated from spot urine samples provides an accurate reflection of 24-h urinary excretion [65]. Subsequently the authors recommend that, whilst spot urines provide a good reflection of the iodine status of a population, 24-h urine samples should be used to determine the iodine intake of a population. In the present study, the mean 24-h UIE, as an indicator of daily iodine intake, of both age groups exceeded the EAR without exceeding the recommended upper level of intake (UL) of 300 μg/day [56].

The iodine intakes determined using 24-h UIE in the present study were 40% lower than the iodine intakes determined using 24-h food recalls in the AHS (i.e., 94 vs. 156 μg/day for 4–8 year olds and 111 vs. 179 μg/day in 9–13 year olds) [59]. As previously discussed, it is not unusual to observe lower iodine intakes in Victorians and this could explain the lower intakes observed in our study compared to the AHS. Furthermore, we utilized 24-h urine samples which are able to provide a more objective measure of actual iodine intake when compared to 24-h food recalls. In addition to the recall and subject biases associated with food recalls [66] food composition databases often lack specificity regarding the use of iodized salt during food production and the salt iodine content [67,68]. Furthermore, the iodine content of dairy products in particular is highly variable and can depend on a number of factors including the breed of cow, supplementation of feed, the use of salt licks, farm location and contamination by iodophore sanitizers during processing [69,70,71]. These variations are often not captured by food composition databases and this can result in inaccurate estimations of iodine intakes when dietary assessment methodologies are used [43].

We also found that actual iodine intake, as measured using UIE, was significantly lower in females, with a higher proportion of female participants having a UIE below the age-specific EAR. To date, ours is the first Australian study to utilize 24-h urine samples to assess differences in iodine intakes, as measured using UIE, between genders. The lower intake of iodine in females is of concern as dietary habits tend to track into adulthood [72] and low iodine intakes during childhood could lead to females entering pregnancy with suboptimal iodine status. Iodine is an important nutrient during pregnancy, and deficiency during this time has been linked with impaired growth and cognition [73,74]. Therefore, it is important that the iodine intakes of schoolchildren, particularly females, continue to be monitored using 24-h urine samples.

The major strength of the current study is the use of 24-h urine samples to objectively measure iodine intake in a relatively large number of Victorian schoolchildren. A stringent 24-h urine collection protocol, specifically tailored to children was used to ensure the completeness of the samples. Limitations of this study include the convenience sampling method, which resulted in the over-representation of participants from a higher socioeconomic background. Regardless of which SES definition was used, almost two-thirds of the participants were from a high socioeconomic background, compared to 28% of 5–11 year olds (based on parental education) in the AHS [75], and this limits our ability to apply our findings to the general population. Finally, as we were limited to a single 24-h urine collection, we were not able to adjust for the within person variation in 24-h urinary iodine excretion to obtain a measure of long-term “usual” iodine intake.

## 5. Conclusions

In conclusion, this is the first study to assess the iodine intakes of Australian schoolchildren post-mandatory fortification of bread with iodized salt using 24-h urine samples. Both daily UIE and UIC determined from 24-h samples indicate that the iodine intakes of this population are sufficient. These results confirm the results of the AHS and indicate that the iodine intakes of Victorian schoolchildren have increased to adequacy since mandatory fortification of bread with iodized salt was introduced in 2009. However, continued monitoring of iodine intakes using 24-h urine samples is needed in order to ensure that the actual iodine intakes of school children are sufficient.

## Figures and Tables

**Table 1:** Descriptive characteristics of participants—mean (SD) or *n* (%).

	All	Boys	Girls
Participants, *n* (%)	650	359 (55)	291 (45)
Age (mean (SD), years)	9.3 (1.8)	9.3 (1.9)	9.2 (1.7)
Age group			
4–8 years, *n* (%)	280 (43)	157 (44)	123 (42)
9–12 years, *n* (%)	370 (57)	202 (56)	168 (58)
SES ^1^			
Bottom tertile, *n* (%)	105 (16)	45 (13)	60 (21) ^a^
Mid tertile, *n* (%)	144 (22)	77 (22)	67 (23)
High tertile, *n* (%)	401 (62)	237 (66) ^a^	164 (56)
SES ^2^			
Bottom tertile, *n* (%)	134 (24)	77 (25)	57 (24)
Mid tertile, *n (%)*	84 (15)	54 (17)	30 (12)
High tertile, *n* (%)	336 (61)	183 (58)	153 (64)
BMI category			
Underweight, *n* (%)	67 (10)	34 (9)	33 (11)
Healthy, *n* (%)	473 (73)	270 (75)	202 (69)
Overweight, *n* (%)	91 (14)	45 (13)	46 (16)
Obese, *n* (%)	20 (3)	10 (3)	10 (4)
Day of urine collection			
School day, *n* (%)	304 (47)	168 (47)	136 (47)
Non-school day, *n* (%)	346 (53)	191 (53)	155 (54)
U-vol (mean (SD), mL/24-h)	873 (424)	897 (453)	843 (384)
U-Cr (mean (SD), mmol/24-h)	5.6 (2.0)	5.8 (2.1) ^b^	5.3 (1.9)
UIE (mean (SD), μg/24-h)	104 (54)	112 (57) ^b^	93 (48)
<EAR *, *n* (%)	177 (27)	77 (21)	100 (34) ^a^
>EAR *, *n* (%)	473 (73)	282 (79) ^a^	191 (66)
UIC (μg/L)			
mean (SD)	133 (68)	141 (70) ^b^	123 (64)
median (IQR)	124 (83,172)	135 (88,180)	116 (75,161)
<100 μg/L, *n* (%)	233 (36)	114 (32)	119 (41) ^a^
<50 μg/L, *n* (%)	49 (8)	20 (6)	29 (10) ^a^

SD: standard deviation; SES: Socioeconomic status; BMI: body mass index; EAR: estimated average requirement; U-vol: urine volume; U-Cr: urinary creatinine; UrNa: UIE: urinary iodine excretion; UIC: urinary iodine concentration; * EAR for 4–8 year olds: 65 μg/day, 9–13 year olds: 75 μg/day [56]; ^1^ SES based on state-based socioeconomic indexes for areas, index of relative socioeconomic disadvantage [48]; ^2^ SES based on primary caregiver education—data not available for all 650 participants, *n* = 554; ^a^ *χ^2^* test *p* < 0.05 for differences between genders; ^b^ Multiple regression adjusted for age and school cluster *p* < 0.05 for difference between genders.

**Table 2:** Urinary parameters by age group (*n* = 650).

	Age Group (Years)	
	4–8 (*n* = 280)	9–12 (*n* = 370)
U-vol (mL/24-h)	749 (341)	966 (456) ^a^
U-Cr (mmol/24-h)	4.3 (1.3)	6.5 (2.0) ^a^
UIE (μg/24-h)	94 (48)	111 (57) ^a^
<EAR *, *n* (%)	80 (29)	97 (26)
>EAR *, *n* (%)	200 (71)	273 (74)
UIC (μg/L)		
mean (SD)	138 (69)	129 (67)
median (IQR)	127 (86,175)	122.3 (80,170)
<100 μg/L, *n* (%)	89 (32)	144 (39)
<50 μg/L, *n* (%)	19 (7)	30 (8)

EAR: estimated average requirement; U-vol: urine volume; U-Cr: urinary creatinine; UIE: urinary iodine excretion; UIC: urinary iodine concentration; * EAR for 4–8 year olds: 65 μg/day, 9–13 year olds: 75 μg/day [56]; ^a^ Linear regression (adjusted for gender and school cluster) *p* < 0.05 for differences between age groups.

**Table 3:** Urinary parameters by school postcode socioeconomic status (*n* = 650).

	SES Category		
	Bottom Tertile (*n* = 105)	Mid Tertile (*n* = 144)	Top Tertile (*n* = 401)
U-vol (mL/24-h)	868 (478)	813 (405)	896 (415)
U-Cr (mmol/24-h)	5.7 (2.1)	5.1 (1.7)	5.7 (2.1)
UIE (μg/24-h)	115 (53)	100 (47)	102 (56)
<EAR *, *n* (%)	20 (20)	38 (26)	119 (30)
>EAR *, *n* (%)	85 (81)	106 (74)	282 (70)
UIC (μg/L)			
mean (SD)	150 (70)	139 (72)	127 (65)
median (IQR)	136 (89,195)	130 (85,175)	121(78,166)
<100 μg/L, *n* (%)	32 (30%)	48 (33)	153 (38)
<50 μg/L, *n* (%)	2 (2%)	11 (8)	36 (9)

EAR: estimated average requirement; U-vol: urine volume; U-Cr: urinary creatinine; UIE: urinary iodine excretion; UIC: urinary iodine concentration; * EAR for 4–8 year olds: 65 μg/day, 9–13 year olds: 75 μg/day [56].
